# Effect of major school playground reconstruction on physical activity and sedentary behaviour: Camden active spaces

**DOI:** 10.1186/s12889-017-4483-5

**Published:** 2017-06-07

**Authors:** Mark Hamer, Daniel Aggio, Georgina Knock, Courtney Kipps, Aparna Shankar, Lee Smith

**Affiliations:** 1 School of Sport, Exercise & Health Sciences, National Centre for Sport & Exercise Medicine, Loughborough University, Leicestershire, UK; 2 Department of Epidemiology & Public Health, University College London, London, UK; 3 Institute Sport, Exercise & Health, University College London Hospital, London, UK; 4 Population Health Research Institute, St. George’s, University of London, London, UK; 5 The Cambridge Centre for Sport and Exercise Sciences, Department of Life Sciences, Anglia Ruskin University, Cambridge, UK

**Keywords:** School, Children, Active play, Quasi-experimental, Physical activity

## Abstract

**Background:**

The physical school environment is a promising setting to increase children’s physical activity although robust evidence is sparse. We examined the effects of major playground reconstruction on physical activity and sedentary time in primary schools using a quasi-experimental design (comparison group pre-test/post-test design).

**Methods:**

Five experimental and two control schools from deprived areas of inner city London were recruited at baseline. Main outcome was physical activity and sedentary time measured from objective monitoring (Actigraph accelerometer) at one year follow up. Pupils’ impressions of the new playground were qualitatively assessed post construction.

**Results:**

A total of 347 pupils (mean age = 8 years, 55% boys; 36% Caucasian) were recruited into the study at baseline; 303 provided valid baseline Actigraph data. Of those, 231 (76%) completed follow-up (*n* = 169 intervention; *n* = 62 control) and 77.4% of the sample recorded at least 4 days of Actigraph wear. In mixed models adjusted for age, sex, ethnicity, ratio activity or sedentary/wear time at baseline, wear time at follow up, and school, no differences were observed in total moderate – vigorous activity (B = −1.4, 95% CI, −7.1, 4.2 min/d), light activity (B = 4.1, 95% CI, −17.9, 26.1), or sedentary time (B = −3.8, 95% CI, −29.2, 21.6 min/d) between groups. There were significant age interactions for sedentary (*p* = 0.002) and light intensity physical activity (*p* = 0.008). We observed significant reductions in total sedentary (−28.0, 95% CI, −1.9, −54.1 min/d, *p* = 0.037) and increases in total light intensity activity (24.6, 95% CI, 0.3, 48.9 min/d, *p* = 0.047) for children aged under 9 yrs. old in the intervention.

**Conclusion:**

Major playground reconstruction had limited effects on physical activity, but reduced sedentary time was observed in younger children. Qualitative data suggested that the children enjoyed the new playgrounds and experienced a perceived positive change in well-being and social interactions.

**Electronic supplementary material:**

The online version of this article (doi:10.1186/s12889-017-4483-5) contains supplementary material, which is available to authorized users.

## Background

Regular participation in physical activity has been associated with positive health markers in young people [1, 2] and also tracks through the life-course [3, 4] thus childhood provides a basis for establishing healthy behaviours. National survey data has suggested that a large proportion of children in the UK do not achieve current physical activity recommendations, [5, 6] and this is particularly apparent in deprived inner city areas where the environment is not conducive to active lives. [7] Observational studies have demonstrated an association between the physical environment (e.g. green space) and levels of physical activity [8] although data from experimental approaches are lacking.

Existing interventions to promote physical activity in children have generally produced small or null effects. [9–11] There is, however, increasing interest to promote young people’s health by ensuring that the school environment supports healthy behaviours [12]. In particular, the physical school environment has attracted interest [12] although there is presently limited robust empirical evidence on the effects of changing the physical environment on activity levels in children. Existing data on the effects of playground design on physical activity have produced mixed findings [13, 14] likely owing to weaknesses in intervention design and lack of long term follow up.

Existing interventions of physical school environments have mostly attempted to modify playground markings and, to the best of our knowledge, only one study investigated the impact of “major” playground reconstruction [15]. This study, performed in north America, used direct observation to assess physical activity during the school day. This method limits the ability to examine carry over effects outside the school environment (ie, at weekends and during evenings), and without objective assessment one cannot tease apart intervention influences on the full spectrum of physical activity intensity, including sedentary time. The aim of the present study was to examine the effects of major playground reconstruction on objective physical activity and sedentary levels in schools recruited from inner city London. We hypothesized that the intervention would promote total daily physical activity and reduce sedentary time, largely at school. The design of playgrounds was facilitated through consultation with children to inform imaginative play environments.

## Methods

### Study design and recruitment

Camden Active Spaces was a school-based quasi-experimental study examining physical activity before (summer term 2014) and after (summer term 2015) major playground reconstruction. The study protocol has been previously published [16] before analysis of any data. The schools were selected by Camden Borough Council based on the highest levels of deprivation (proportion of free school meals) and local area-level data on childhood obesity. Researchers attended assemblies in April/May 2014 to disseminate information on Camden Active Spaces, and children were given participant study information sheets. In order to make parents aware of the study an information sheet was distributed to them (translated into different languages where required). Head teachers from each school provided explicit written consent for their schools and school children to take part in the study. Parents were given the option to “opt-out” their child(ren). The study was presented as voluntary and children were free to withdraw at any time. Ethical approval was granted by the University College London Research Ethics Committee (4400/002).

### Intervention

**Fig. 1:**
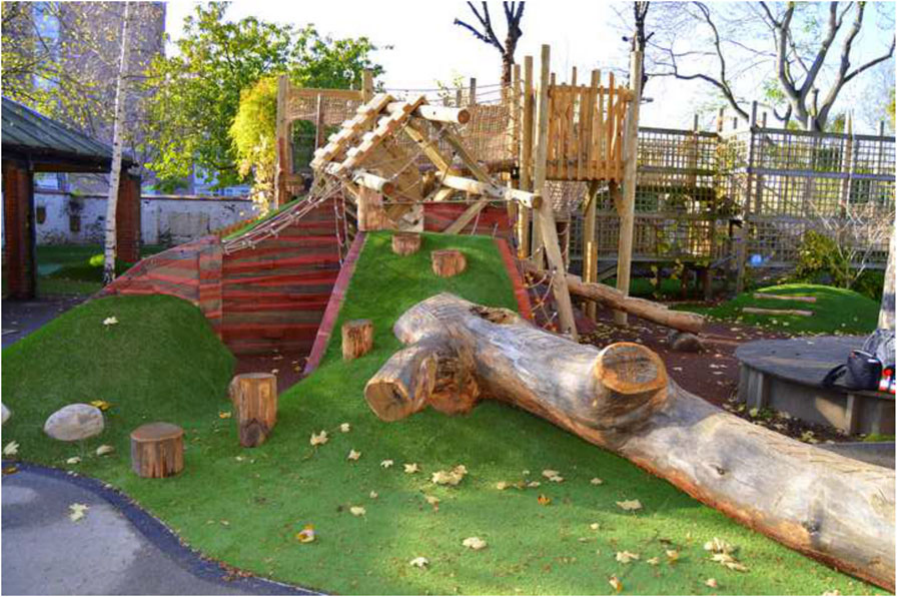
A new playground construction in the Camden Active Spaces project

Camden Borough Council appointed two design teams through competitive tender to re-design existing school playgrounds (five primary schools and two secondary schools). However, as appropriate controls for secondary schools could not be identified the present analyses focused only on primary schools. The design teams undertook consultations with teachers and children from each school in order to inform their designs. The primary goal was to design playground areas conducive to physical activity via active play, with bespoke features to engage children to become more active. Each school received a unique playground design, for example displayed in Fig. 1. Unique features included new AstroTurf games pitches, climbing frames, trampolines, monkey bars, and outdoor gyms, which were designed based on themes emerging from consultations (e.g. ancient ruins, volcanoes, clouds etc.). The research team did not provide input into the design of the playgrounds. Building work started in August 2014 and new playgrounds were completed by December 2014 in all schools.

### Primary outcome: Physical activity assessment

Trained researchers fitted accelerometers (waist mounted Actigraph GT3X) to children during the school day. Children were asked to wear the device during waking hours every day for seven consecutive days, but not during water-based activities or sleep. Devices were programmed to sample at 30 Hz. Our protocol followed methods used in the International Childrens’ Accelerometry Database study [2]. Briefly, data files were reintegrated to a 60-s epoch and none wear time was defined as 60 min of consecutive zeros, allowing for 2 min of none zero interruptions. The first partial day of wear was excluded from our analyses in order to reduce the possibility of reactivity to wearing the device (ie, increased physical activity driven by novelty effect). All children with at least 1 school day and at least 500 min of measured monitor wear time between 07:00 AM and midnight were included. Total physical activity was expressed as total counts, including sedentary minutes, divided by measured time per day (counts/min, cpm). Time spent sedentary was defined as all minutes less than 100 cpm, light activity from 100 up to 3000 cpm, and moderate-vigorous physical activity (MVPA) as more than 3000 cpm. In an attempt to maximise response rates and adherence to protocol, each child who completed the wear protocol was awarded a one-month swimming voucher and entered into a prize draw to win an iPod Touch. All schools taking part in the study were entered into a separate prize draw to win one of two Nintendo Wiis.

### Secondary outcomes

Weight and body composition were measured using the Tanita SC-330 Body Composition Analyser (Tanita Inc., IL, USA) in light clothing, and height was measured using the Leicester Height measure with participants in the Frankfort plane. Body mass index (BMI) was calculated from weight (kg)/height squared (m^2^). Four fitness tests were carried out: grip strength was assessed from the dominant hand using a hand held Dynamometer; the standing horizontal jump test was performed to assess leg strength; peak flow was measured using a peak flow meter to assess lung function; and the sit-and-reach test to assess flexibility. All tests were performed three times and the highest recording was used for analyses.

### Covariates

Participants’ age (grouped as <9 years and 9–10 years), sex, and ethnic background (Caucasian, Mixed, Asian, Black, Other) was self-reported although children were supervised by the researchers and assistance provided where necessary.

### Process evaluation

A process evaluation was carried out one year post construction of the new playground. The evaluation aimed to explore children’s playground engagement; from children’s, parent’s and teacher’s accounts of their experiences (see Additional file 1: Table S1 for topic guide). At two intervention schools, semi-structured focus groups with 12 children (6 from each school) and face-to-face individual interviews with two teachers and two parents were carried out. Audio recordings were transcribed verbatim.

### Analyses

Differences in baseline characteristics between control and intervention schools were examined using independent samples T-tests (*p* < 0.05 denoted as significance level). Mixed models, adjusted for age, sex, ethnicity, the ratio of activity/wear time at baseline, wear time at follow up (as fixed effects), and school (as random effect, to account for clustering at school level) were employed to compare physical activity (MVPA and light activity) at follow up between intervention and control. We also examined sedentary time as the outcome, but for these analyses we adjusted for the ratio of baseline sedentary/wear time instead. We examined the physical activity/sedentary outcomes separately over the standard school day (09:00–15.00) and also for the total day (07:00–00:00) (including weekends). As a post hoc analysis, an age interaction term (binary variable: <9 yrs./9–10 yrs) was fitted to the model. All analyses were conducted using SPSS version 22 with statistical significance as *p* < 0.05.

Thematic analysis, a qualitative method for identifying, analysing, and reporting themes, was used to analyse focus group and interview data. Thematic analysis was chosen to provide a rich description of the data and to identify themes at an explicit level using a realist approach [17]. Transcripts were reviewed independently by two researchers (GK, LS) who each generated an initial list of codes. These lists were then amended and refined through discussion until a single list was agreed. All transcripts were coded and entered into NVivo version 10 (QSR International Pty Ltd., 2012). Once the coding had been agreed, the coded transcripts were reviewed to search for common themes specifically related to children’s playground engagement.

## Results

### Baseline characteristics

A total of 347 participants from 5 intervention and 2 control schools were recruited into the study at baseline. Valid baseline Actigraph data were provided in 303 children, and of those, 231 (76%) completed follow-up. Reasons for drop-out included left school/absent on day of follow up data collection (*n* = 14), refusal to wear accelerometer at follow up (*n* = 12), insufficient wear time (*n* = 21), and failure to return device (*n* = 25). There were no differences in drop-out between control and intervention groups (17.3% vs. 25.8%, *p* = 0.13), and no other significant differences in characteristics (age, sex, BMI, ethnicity) between drop-outs and the final analytic sample were observed.

Table 1 displays the baseline characteristics of participants in control and intervention groups. In the overall sample, 77.4% recorded at least 4 days of Actigraph wear and 7.4% only 1 day of wear at baseline. The groups were largely similar except for slight differences in ethnic distribution (greater proportion of Asian and Black children in control), and Actigraph wear time. In models adjusted for wear time, total MVPA at baseline did not significantly differ between groups (B = 4.1 min/d, 95% CI, −0.8, 8.9, *p* = 0.10), although total sedentary time was higher in the control group (B = 41.5 min/d, 95% CI, 25.4, 57.5, *p* = 0.001) and light activity lower (B = −27.9 min/d, 95% CI, −42.3, −13.5, *p* = 0.001) compared to intervention.

**Table 1:** Baseline characteristics of the sample

Variable	Control (*n* = 62)	Intervention (*n* = 169)	*p*-value
Age distribution (%)			
< 9 yrs	30 (48.4)	95 (56.2)	0.29
9–10 yrs	32 (51.6)	74 (43.7)	
Sex (%)			
Female	33 (53.2)	79 (46.7)	0.38
Male	29 (46.8)	90 (53.2)	
Ethnicity (%)			
Caucasian	20 (32.2)	60 (35.5)	0.03
Mixed	5 (8.1)	24 (14.2)	
Asian	15 (24.2)	23 (13.6)	
Black	16 (25.8)	26 (15.4)	
Other	6 (9.7)	36 (21.3)	
Body mass index (kg/m^2^)	17.3 ± 2.9	17.5 ± 3.4	0.63
Body fat (%)	22.6 ± 7.0	21.5 ± 7.4	0.30
Hand grip (kg)	13.4 ± 3.8	13.0 ± 4.7	0.52
Sit and reach (cm)	23.2 ± 5.8	23.2 ± 16.9	0.98
Horizontal jump (cm)	110.4 ± 18.5	106.8 ± 20.7	0.23
Peak flow (l/min)^a^	204.9 ± 41.9	154.0 ± 55.7	0.001
Actigraph wear time (min/d)	715.2 ± 85.9	755.9 ± 83.9	0.001
Valid wear days	4.3 ± 1.5	4.7 ± 1.7	0.14
Total MVPA (min/d)	24.8 ± 12.3	30.1 ± 17.5	0.03
Total light activity (min/d)	337.6 ± 70.9	392.2 ± 56.9	0.001
Total sedentary (min/d)	352.7 ± 55.9	333.6 ± 75.4	0.07
School time MVPA (min/d)	14.1 ± 7.6	13.6 ± 7.5	0.69
School light activity (min/d)	158.7 ± 29.5	178.1 ± 31.1	0.001
School time sedentary (min/d)	187.2 ± 32.3	168.3 ± 32.2	0.001

Data presented as mean ± SD unless stated

*MVPA* Moderate to vigorous physical activity

^a^age and height adjusted

### Effects of intervention

In mixed models no differences were observed in physical activity or sedentary time between control and intervention over the whole day (Table 2) or specifically during school time (Table 3).

**Table 2:** Physical activity and sedentary time (total day: 07:00–00:00) at one year follow up in the intervention children compared to control

	Full sample	Under 9 yr. olds	9–10 yr. olds
*Sedentary (min/d)*			
Control	Ref	Ref	Ref
Intervention	−3.8 (−29.2, 21.6)	−28.0 (−1.9, −54.1)	18.7 (−20.6, 57.9)
*Light PA (min/d)*			
Control	Ref	Ref	Ref
Intervention	4.1 (−17.9, 26.1)	24.6 (0.3, 48.9)	−13.1 (−46.2, 20.1)
*MVPA (min/d)*			
Control	Ref	Ref	Ref
Intervention	−1.4 (−7.1, 4.2)	3.5 (−3.0, 10.0)	−7.7 (−18.3, 2.9)
*Total cpm*			
Control	Ref	Ref	Ref
Intervention	−5.5 (−119.9, 108.9)	109.1 (−2.8, 221.0)	−135.8 (−344.6, 73.1)

Coefficients (95% CI) adjusted for age, sex, ethnicity, ratio activity or sedentary/wear time at baseline, wear time at follow up (as fixed effects), and school (as random effect)

**Table 3:** Physical activity and sedentary time (school day: 09:00–15:00) at one year follow up in the intervention children compared to control

	Full sample	Under 9 yr. olds	9–10 yr. olds
*Sedentary (min/d)*			
Control	Ref	Ref	Ref
Intervention	−6.8 (−38.8, 25.2)	−19.0 (−1.0, −37.0)	11.2 (−33.8, 56.4)
*Light PA (min/d)*			
Control	Ref	Ref	Ref
Intervention	6.9 (−19.4, 33.1)	17.1 (0.1, 34.2)	−7.1 (−46.6, 32.5)
*MVPA (min/d)*			
Control	Ref	Ref	Ref
Intervention	−0.8 (−7.1, 5.5)	1.6 (−2.4, 5.6)	−5.0 (−10.5, 0.5)
*Total cpm*			
Control	Ref	Ref	Ref
Intervention	−5.5 (−119.9, 108.9)	109.1 (−2.8, 221.0)	−135.8 (−344.6, 73.1)

Coefficients (95% CI) adjusted for age, sex, ethnicity, ratio activity or sedentary/wear time at baseline, wear time at follow up (as fixed effects), and school (as random effect)

In post-hoc analyses there were significant age interactions for sedentary (*p* = 0.002) and light intensity physical activity (*p* = 0.008). Compared to control, children under 9 yrs. of age in the intervention group demonstrated reductions in total sedentary time (−28.0, 95% CI, −1.9, −54.1 min/d, *p* = 0.037) and increases in total light intensity physical activity at follow up (24.6, 95% CI, 0.3, 48.9 min/d, *p* = 0.047), although no effects were observed in older children (aged 9–10 yrs) (Table 2). These effects were also seen over the school day (Table 3).

In sensitivity analyses we re-processed all data using 5-s epochs in an attempt to uncover sporadic bursts of activity that could have been smoothed out by the longer 60 s epoch employed. However the results were not appreciably changed (data not shown). In a further analysis we re-run the models after removing children with only one day of Actigraph wear although results did not change.

No main effects or age interactions were observed for any of the secondary outcomes (data not shown).

### Process evaluation

Three primary themes were identified (see Additional file 1: Table S2), ordered by prevalence: 1) enjoyment, 2) perceived changes in well-being, 3) social interactions. Extracted quotes are provided in Additional file 1: Table S3.

## Discussion

This quasi-experimental study is the first to assess the effects of major playground reconstruction in UK schools on objectively assessed physical activity and sedentary time. At one year follow up we found reductions in sedentary time that was displaced by increased light intensity activity in younger children under 9 years of age. There were no effects on MVPA or any secondary outcomes.

### Strengths and limitations

It was not feasible to randomise schools although control and intervention children were largely comparable in terms of socio-demographic and physical variables (such as BMI and fitness data). Indeed, randomised controlled trial designs are more suited to interventions targeted at the individual as oppose to natural experiments. Based on our prior calculations [16] the final sample size was underpowered to detect small changes in MVPA, although such effects are unlikely to be clinically meaningful. No consensus currently exists regarding appropriate cut points in children’s accelerometry studies [18] thus we chose to use a conservative cut point to derive MVPA. Some moderate intensity activity could therefore have been misclassified as light activity. Nevertheless, the low proportion of children meeting the physical activity guideline in the present study is consistent with data from a prior survey using self report where only 12% of children from Camden met the guideline [19]. The experimental rigour of the present study was maximised by employing a longitudinal design, performing a comparison with control schools, implementing a robust objective measurement of physical activity (avoiding seasonal effects by performing baseline and follow-up at the same time of year), and employing an ethnically diverse sample to increase generalisability of the findings. The playgrounds were designed by professional design teams informed by the children, and assessed independently by researchers.

### Comparison with other studies

School-based interventions, such as printed educational materials and changes to the school curriculum, have had limited effects on physical activity levels and sedentary behaviour [9–11]. Other interventions have focused on maximising physical activity during recess where children are free to choose their activities. Existing evidence has suggested that social structures, physical ability and playground space may influence physical activity levels during recess [20] For example, restricting activities that dominate the playground (ie, soccer played by strongest boys) to specified areas or allowing fewer children at the same time to play have been trialled as strategies to increase physical activity [21, 22]. Other interventions have included playground markings [23–25] time-management [26] obstacle courses or fitness breaks, [27] equipment provision and increasing the amount of playground facilities [28–31] and combinations of these approaches [32–34]. Taken together these studies have produced mixed findings possibly because of short-term follow up, weak study design (e.g., lack of control groups), and some without objective physical activity assessment. Nevertheless, they provide a possible explanation as to why the present intervention was successful only in the younger children, as the new playground structures are likely to have created dedicated space for younger, more timid children to play (ie, play on new equipment was restricted to certain classes on each day) and restricted other more dominant activities to certain areas of the playground.

Consistent with other interventions that have employed objective physical activity assessment [10] we did not observe any changes in MVPA, only increases in light intensity activity that displaced sedentary time. This is perhaps unsurprising as the types of moderate -vigorous activities undertaken on the new playground structures (e.g. climbing and swinging) may not have been properly recorded by the accelerometer. This type of activity may foster improvements in muscle strength and balance, and it is possible that the full impacts of the intervention on physical health were under-estimated. Despite large changes in sedentary time we did not observe effects on secondary outcomes such as adiposity, which is largely consistent with existing literature where high quality evidence on the adverse health effects of sedentary behaviour in children is lacking [35–37]. Stronger evidence on health effects of sedentary behaviour exists in adult populations [38, 39] and sedentary habits are likely to track across the lifecourse [4] thus reducing sedentary time in childhood may yield more active behaviours in adulthood.

Existing playground interventions have tended to only measure physical activity during recess periods although we measured total activity/sedentary across the whole day (on school days and the weekend) as the children may have compensated for any increase in physical activity at school by increases in sedentary time after school. In fact our data suggested that the reduction in school sedentary time only reflected ~70% of the reduction in total sedentary time. Some schools allowed children to use the new playgrounds after school. Nevertheless, the correlates of after school sedentary behaviour are poorly understood [40].

### Process evaluation

The process evaluation suggests that the new playgrounds were enjoyed by the children using them and that this had positive repercussions on perceived self-efficacy, well-being and social interactions. This is of interest as research suggests that self-efficacy and social interaction have a positive association with children’s health behaviours [41]. Indeed, an increase in self-efficacy has been shown to increase physical activity participation [42].

## Conclusion

Changing the physical school environment did not influence physical activity. However, in post-hoc analyses the intervention was effective in displacing sedentary time in younger children only. The intervention was unsuccessful in older children suggesting that more intensive interventions are required involving not only the physical environment but also at the level of the individual, the family, and societal levels. Qualitative data suggested that the children enjoyed the new playgrounds and experienced a perceived positive change in well-being and social interactions.

## Additional file

Additional file 1: Results from the qualitative study (**Table S1.** Topic Guide; **Table S2.** Overview of thematic results; **Table S3.** Extracted quotes) (DOCX 17 kb).

## Abbreviations

BMI: Body mass index
MVPA: Moderate-vigorous physical activity
