# Supplementary material for: Effect of major school playground reconstruction on physical activity and sedentary behaviour: Camden active spaces
Source: BMC Public Health. 2017 Jun 7;17:552. doi: 10.1186/s12889-017-4483-5 (PMC5463303; doi:10.1186/s12889-017-4483-5)
Supplement: Additional file 1: — Results from the qualitative study (Table S1. Topic Guide; Table S2. Overview of thematic results; Table S3. Extracted quotes) (DOCX 17 kb). [file 12889_2017_4483_MOESM1_ESM.docx]

**Supplementary Tables**

**Table S1.** Topic Guide

**Likes/Dislikes**

1. What do you think about the new playground?

2. What do you like about being outside?

3. What is the feature in the new playground you play on the most and why?

4. Did you like being involved with the design of the new playground and why? Was there a certain method you enjoyed?

**Enjoyment in physical activity**

1. Compared to before, how do you use parts of the playground differently now?

2. Do you think your activity levels changed in the new playground? – do you like being active?

3. How do you think being active effects your health? (Possibly use visual aids to help define health)

4. Do you spend more time outside since the new playground has been built?

5. Are you more active outside school since the new playground? For example, play more football/chase with your friends?

6. Would you use the playground after school and in school holidays?

7. Has the new playground changed how you commute to school?

*Parents/Teachers:* How has the new playground made you think about your children’s activity levels outside school? Would you supervise them outside school time and during holidays?

**Social interactions**

1. Do you spend more time with your friends in the new playground?

2. Do you play more games with your friends in the new playground?

*Parents/Teachers:* How do you think the new playground has affected the community?

**Well-being**

1. Has your mood changed since the new playground was built? Do you feel happier?

2. *Parents/Teachers*: After playing on the playground at break or Lunch, is there a difference in their concentration levels?

*Parents/Teachers:* What has been different in the children’s behaviour in class/at home since the new playground has been built? For example, change in mood, more sociable and concentration levels.

**Table S2.** Overview of thematic results

| **Theme** | **Sub-theme** | **Description** |
| --- | --- | --- |
| Enjoyment | Duration | The amount of time spent being physically active |
|  | Capabilities | The changes in skills and practical ability when being physically activite |
|  | Understanding of PA | The knowledge of what physical activity does to our physical and mental health |
|  | Types of PA | The types, groups or forms of physical activity children are engaged with in the redesigned playground |
|  |  |  |
| Changes in wellbeing | Self esteem | The redesigned playground effecting an individual's sense of his or her value or worth |
|  | Enjoyment, pleasure, mood | The positive feelings felt from the redesigned playground |
|  | Creativity | The creative activities stemming from the redesigned playground |
|  |  |  |
| Social interactions | Formation and strengthening of relationships with other children | The social interactions between the children which involve new relationships / existing ones enhanced from the redesigned playground |
|  | Conflict resolution | The way the redesigned playground allows conflicts to be resolved between individuals |
|  |  |  |

**Table S3.** Extracted quotes

**Theme 1: Enjoyment**

The first primary theme was evident across all participants through the duration the children spent in the playground:

Parent1: “. . .how do you call that thing in there (points to the top of the palm of her hand)” Moderator: “Calluses? Hard Skin?” Parent: “Yeah, hard skin, really really hard skin in there yeah, on both hands! She (her daughter) gets blisters because she doesn’t stop so it goes away, come back, goes away, comes back, she loves them”

This is added to by the amount of multiple types of PA the children can execute from the design features in the redesigned playground:

"There are such different things to do, she has a variety" Parent 1

“It’s fun, there are more activities” Child school A

“So this is higher, harder, more challenging and of course it’s building upper body strength which a lot of things do not do" Teacher, School A

Furthermore, the children’s physical capabilities and practical abilities are being improved:

"She never use to be able to hold on probably, but now she can go through all of them. So her muscles are stronger, her hands are stronger, it helps with her physical power so it’s good yeah . . .she has stronger muscles now and can hold on longer!” Parent 1

“This is a good alternative (than Physical Education lesson (P.E.)). It develops different things. It develops their different key skills” Teacher, School B

Plus, there has been changes in the children’s knowledge and understanding of PA:

“. . . because if you just sat down on a bench, the whole time, and you just ate junk food, like pizza. . . you won't be as active and you might be, erm , like, a bit broken, kind of. . . I mean like you're going to walk very slowly and you’ll be a very slow person, and you might die a young age" Child, School B

“You can lose weight and be more skinny and flexible” Child, School A

“Being active makes you happy” Child, School B

“It (PA) is just important in general, especially for kids, it just, they’re just more happier, more...yeah they’re just happier humans" Parent 2.

**Theme 2: Changes in Well-being**

This theme included the highest amount of references and was commented on by the majority of the sources. The children’s enjoyment, pleasure, and mood enhanced by the playground is illustrated:

"Sometimes you don’t want to come to school because... I’m not trying to say it’s boring but like, is it quite fun sometimes, and they would come to have playtime and lunchtime but we use to be really

bored of our playground but now it’s really fun and everything, they would come to school every day!" Child, school A

“Yeah our playground it a lot of fun! Thank you for building it!” Child, school A

"Everyone is having a really fun time on the playground" Child, school B

The feeling ‘happiness’ was commented on regularly:

“Being active makes you happy” Child, school B

"She has fun and enjoys it a lot. . . she is much happier" Parent 1

This seemed to equate to a sense of pride felt by the children:

“. . .because you get to run around more and get healthier, have fun and play with your friends, it’s all so many things, all in one because of the new playground, isn’t that great?” Child, school A

“It represents our School better” Child, school A

In turn, from the enjoyment felt from being active in the redesigned playground, it allows the children to feel comfortable to test and build confidence in their abilities which attributes to their self-esteem:

“I suppose what I like most of all about it, is the risk taking requirement of it, a lot of it the slide is good and high, the monkey bars are not easy, the trampoline is quite thrilling actually, you know, we’ve all been on it, adults and children, so it’s hard for them and definitely not easy” Teacher 1

“You can see the younger ones testing themselves more” Child, school A

Also, there is signs of creativity performed:

“'The difference with the playground is that everything is at their own instigation, it’s their choice and they organise the games” Teacher 1

“. . .we make new games because of it” Child, school A

**Theme 3: social interactions**

The changes in well-being may increase the likelihood of formation and strengthening of relationships with other children in the redesigned playground:

“. . .because the playground is so, so exciting it’s been encouraging them to play all as one group or just to have children be a bit more aware of anybody who is being excluded” Teacher 1

"Peoples involving me in their games more which makes me feel very happy" Child, school A

“Everyone is more involved in the games" Child, school A

"We make new friends and play with people we haven’t played with before" Child, school A

"We play with others that we don’t normally play with" Child, school A

This provides opportunities for conflict resolution:

"Sometimes you play with people you really don’t like. . . like there’s an annoying boy in the class and then you play with him and it’s actually really fun" Child, school A

"There’s less gossip because then like there is no more children just walking around and talking and now there is more space to just be free" Child, school A
